# Optimized reconstruction of the crystallographic orientation density function based on a reduced set of orientations

**DOI:** 10.1107/S1600576719017138

**Published:** 2020-02-01

**Authors:** Abhishek Biswas, Napat Vajragupta, Ralf Hielscher, Alexander Hartmaier

**Affiliations:** a Interdisciplinary Centre for Advanced Materials Simulation, Ruhr University, 44801 Bochum, Germany; b Chair of Applied Functional Analysis, Chemnitz University of Technology, 09126 Chemnitz, Germany

**Keywords:** texture reconstruction, integer approximation, crystal plasticity, micromechanical modeling

## Abstract

Crystallographic textures, as they develop for example during cold forming, can have a significant influence on the mechanical properties of metals, such as plastic anisotropy. Textures are typically characterized by a non-uniform distribution of crystallographic orientations that can be measured by diffraction experiments like electron backscatter diffraction (EBSD). Such experimental data usually contain a large number of data points, which must be significantly reduced to be used for numerical modeling. However, the challenge in such data reduction is to preserve the important characteristics of the experimental data, while reducing the volume and preserving the computational efficiency of the numerical model. For example, in micromechanical modeling, representative volume elements (RVEs) of the real microstructure are generated and the mechanical properties of these RVEs are studied by the crystal plasticity finite element method. In this work, a new method is developed for extracting a reduced set of orientations from EBSD data containing a large number of orientations. This approach is based on the established integer approximation method and it minimizes its shortcomings. Furthermore, the *L* ^1^ norm is applied as an error function; this is commonly used in texture analysis for quantitative assessment of the degree of approximation and can be used to control the convergence behavior. The method is tested on four experimental data sets to demonstrate its capabilities. This new method for the purposeful reduction of a set of orientations into equally weighted orientations is not only suitable for numerical simulation but also shows improvement in results in comparison with other available methods.

## Abstract 2

In this work, a new method is developed to reconstruct the orientation distribution function from experimental data with a set of equally weighted orientations. It is based on the deterministic integer approximation method, but it minimizes the shortcomings of this method by optimizing the orientation grid and kernel function.

## 1. Introduction   

Microstructure characterization of polycrystalline material performed using diffraction experiments provides information regarding the crystallographic orientations. Depending on the resolution and the size of the scanned area, a diffraction experiment like electron backscatter diffraction (EBSD) may consist of thousands and sometimes millions of measurement points of crystallographic orientations. A non-uniform distribution of these crystallographic orientations is represented with the help of a probability density function commonly referred to as the orientation density function (ODF) (Kocks *et al.*, 2000 ▸). The ODF can then be estimated by superposition of radially symmetric kernel functions centered at each crystallographic orientation (Hielscher, 2013 ▸).

Texture plays an important role in material behavior and is mainly responsible for anisotropy (Hielscher *et al.*, 2014 ▸). Numerical predictions of the anisotropic mechanical response in metals have been of special interest. In view of this, many macroscopic numerical models have been proposed [a few examples of such models are given by Hill (1948 ▸) and Barlat *et al.* (1991 ▸)]. However, these models lack a clear connection to the important microstructural features like texture, grain size *etc*. that influence the mechanical response. Micromechanical modeling approaches address these limitations by incorporating the important microstructural features into the numerical model.

To properly characterize the crystallographic orientations of grains in a polycrystalline material, EBSD measurements are usually performed with high spatial resolution (Humphreys, 2004 ▸). This inevitably results in a large number of measurement points. For micromechanical modeling of polycrystalline materials in the crystal plasticity finite element method (CPFEM) framework (Roters *et al.*, 2010 ▸), an optimal number of orientations that closely represent the material characteristics are required. There are two main approaches for modeling polycrystals. In the first approach the grain volume fractions are considered equal and fixed, which requires a set of equally weighted orientations. In this regard, various ODF reconstruction strategies have been proposed. One such method is the ‘hybrid integer approximation’, which has been introduced for discrete ODFs by Eisenlohr & Roters (2008 ▸). This method combines the probabilistic sampling approach of Tóth & Houtte (1992 ▸) with the deterministic integer approximation of Leffers & Jensen (1986 ▸).

In the second modeling approach, grain volume statistical distributions are considered, and thus the reconstruction of the ODF is done by a set of weighted orientations. Recent work by Schaeben *et al.* (2017 ▸) introduces such a method, which utilizes the Dirichlet kernel to provide an unbiased estimate of the Fourier coefficient up to any finite order. The corresponding weights are estimated numerically and can be related to the Fourier transform due to the linearity of both the spatial and spectral domains. Further information about other reconstruction methods can be found in the work of Eisenlohr & Roters (2008 ▸), Schaeben *et al.* (2017 ▸) and references therein.

In the following, we introduce a method for reconstructing the ODF estimated from a given set of experimentally measured orientations by a smaller number of equally weighted orientations (sample orientations). The method begins with an initial guess of an equi-spaced SO(3) grid. The experimentally measured orientations are superimposed on this grid. The orientations lying within a distance of half grid spacing from a given grid point are defined as a cluster around that grid point. By utilizing an iterative scheme based on the work of Leffers & Jensen (1986 ▸), the number of required sample orientations is divided among the grid points in proportion to the size of the cluster of input orientations and it is represented by an integer value. Any cluster of orientations around a grid point with an associated integer value greater than one is further divided into smaller groups of orientations from which mean orientations are sampled. These mean orientations collectively form the reduced set of orientations. The ODF is estimated from the reduced set of orientations by employing a kernel function with an optimized shape parameter. The error function is the *L* ^1^ norm of the difference between the ODFs from the experiments and the reduced set of orientations. This process is continued iteratively with the intention of minimizing the error function by optimizing the SO(3) grid. This algorithm is implemented using the *MTEX* (Bachmann *et al.*, 2010 ▸) toolbox in MATLAB.

The capability of this method is demonstrated by implementing it on three cubic crystal cases in the form of cold-rolled OFHC copper (Rolled-Cu) (Anand, 2004 ▸), additively manufactured 316L stainless steel produced by two different laser powers – 1000 W (316L-1000W) and 400 W (316L-400W) (Biswas *et al.*, 2019 ▸) – and one orthorhombic case, forsterite, which is available as an EBSD example in *MTEX* (Bachmann *et al.*, 2010 ▸). The orientations for Rolled-Cu are generated by plane strain compression simulation as suggested in the work of Anand (2004 ▸) and by extracting the grain orientations at every integration point in the last time step. The simulation is then repeated five times with different sets of random input orientations and the results from all simulations are combined to obtain 27 000 orientations.

## 2. Method   

### 2.1. Problem setup   

Estimation of an ODF 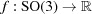 from crystallographic orientation measurements 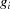, 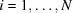, is a classical problem in crystallographic texture analysis (see Table 1 ▸ for notation). The most common method is called kernel density estimation (*cf*. Hielscher, 2013 ▸) and uses a kernel function 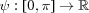 to estimate an ODF as
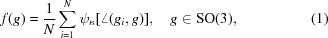
where 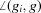 denotes the disorientation angle between the orientations 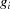 and *g*. Note that the estimated ODF *f* heavily depends on the choice of the kernel function ψ. Here we restrict ourselves to the de la Vallée Poussin kernel 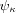 (*cf*. Schaeben, 1997 ▸), the half-width of which can be controlled by the parameter 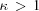.

The objective in this work is to find for a given *N* a much smaller number 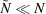 of orientations 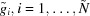, and a kernel parameter 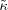 such that the ODF
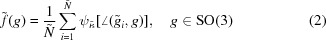
is a good approximation of the ODF *f* estimated from the full data set, *i.e.* 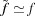. As an error measure we consider the *L* ^1^ norm, which has been used previously in texture analysis by Schaeben *et al.* (2017 ▸) and Bozzolo *et al.* (2007 ▸).
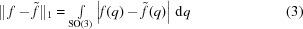
measures the volume of differently oriented orientations between the initially estimated ODF *f* and its approximation 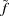. In the extreme case when the ODFs *f* and 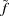 are concentrated around disjoint orientations the error approaches 2. On the other hand, when 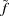 approximates *f* better the error approaches zero.

### 2.2. Outline of the algorithm   

The idea of our algorithm can be summarized in the following steps:

(1) Estimate an initial ODF *f* from the input orientations 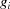, 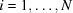, using a fixed kernel function 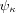 (Section 2.1).

(2) Fix a subdivision of the orientation space into *M* cells with resolution 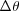 (Section 2.3).

(3) Determine the numbers 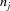, 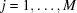, of input orientations 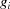, 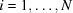, that fall into each of the *M* cells (Section 2.3).

(4) Find a scaling factor α (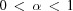) such that the integers 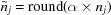 satisfy the condition 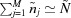, where 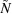 is the target number of orientations of the reduced set (Section 2.4).

(5) For each cell 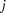, 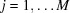, randomly subdivide the corresponding input orientations 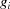, 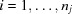, into 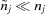 groups and compute the mean orientations 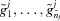 corresponding to each group (Section 2.5).

(6) Compute an ODF 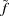 from the reduced set of orientations 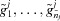, 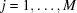, using a kernel function 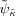 (Section 2.1).

(7) Optimize the kernel function shape parameter 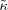 such that the misfit 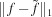 is minimized (Section 2.6).

(8) Repeat steps 2 to 7 for different subdivisions of the orientation space and find the optimal resolution 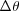 with respect to the final misfit 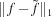 (Section 2.7).

### 2.3. Subdivision of the input orientations   

**Figure 1:**
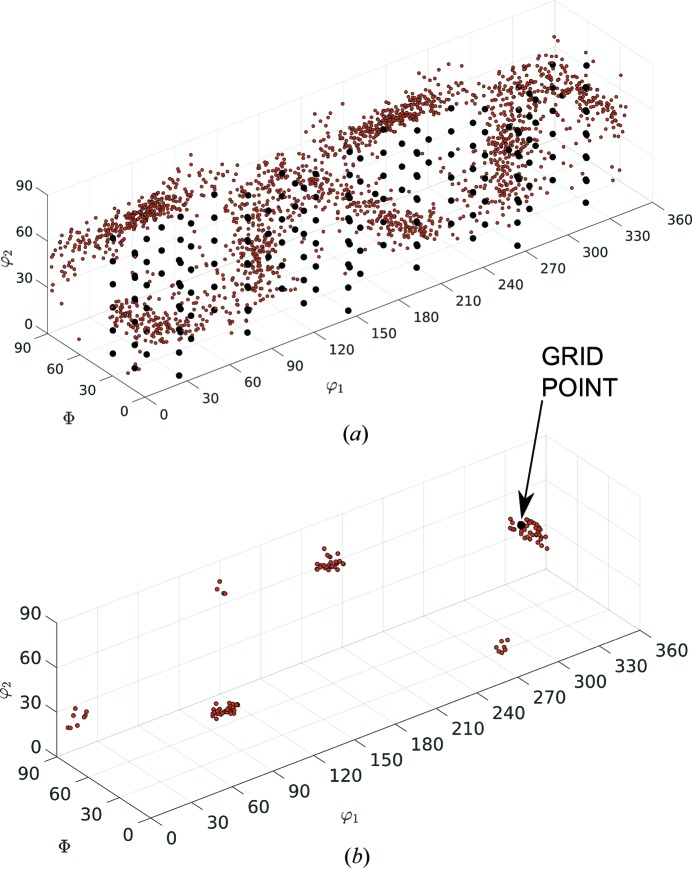
(*a*) Input orientations (red dots) on an SO(3) grid (black dots) for the test case of Rolled-Cu, with grid points with resolution 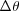 = 15°; here 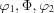 are the Bunge Euler angles. (*b*) An individual grid point with corresponding input orientations within a distance of 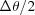 for the case *n* = 117.

The process begins with fixing a resolution 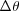 and a corresponding decomposition of the Euler angle space into *M* cells of approximately equal volumes corresponding to each grid point (*cf*. Fig. 1 ▸). Next, we count the number 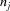, 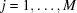, of orientations that fall into each of these cells. An illustration of this process is shown in Fig. 1 ▸ for the test case of Rolled-Cu orientations. For ease of visualization, a grid spacing of 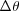 = 15° is selected. The equi-spaced SO(3) is represented by black dots and the input orientations by red dots.

### 2.4. Integer approximation   

In the second step of this algorithm, the target number of samples 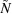 is distributed among the cells in proportion to their counts 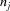, 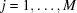. This step is based on the work of Eisenlohr & Roters (2008 ▸), which utilized the original idea of Leffers & Jensen (1986 ▸). It is referred to as ‘integer approximation (IA)’ in the work of Eisenlohr & Roters (2008 ▸). Henceforth, we will use this term accordingly. This iterative scheme estimates a scaling factor α, using a binary search algorithm as suggested by Eisenlohr & Roters (2008 ▸), which is then multiplied with the counts 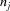 and rounded to the closest integer:
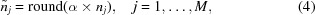
subject to the condition that
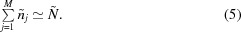



### 2.5. Determination of the downsampling   

The IA provides an integer number 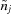 of orientations that need to be computed for each subdivision cell. The objective of this step is to reduce the 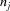 orientations in each cell to only 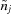 orientations such that the mean of the new orientations is similar to the mean of the original orientations. Hence, if 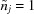, we simply take the mean of all orientations within the cell as the new orientation 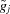.

For cases with 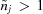, we subdivide the original orientations 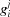, 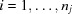, within each cell 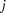, 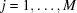, into 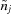 clusters and compute the new orientations 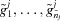 as the mean orientations of each of the clusters. The subdivision is performed randomly; other non-random subdivision methods like sequential sampling were also tested for all of the test cases, but the error 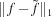 in all cases varied within 1%. To keep the subdivision process unbiased, the random method is chosen.

This is visualized in Fig. 1 ▸, in which a grid point is shown with a cluster of orientations (red dots). In this case, there are 117 orientations in the cell; if we suppose that the corresponding 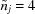, then these orientations are randomly subdivided into four approximately equal clusters containing 29, 30, 29, 29 orientations. The 117 original orientations are then approximated by the 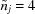 mean orientations of each cluster.

**Figure 2:**
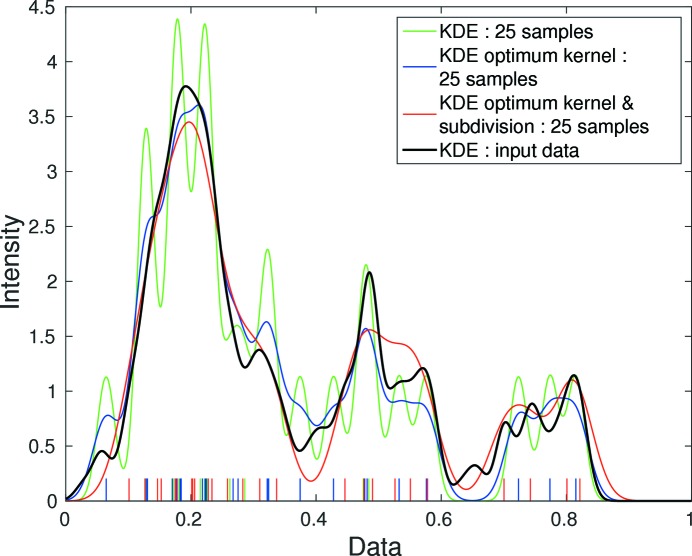
Kernel density estimate (KDE) of the 1D example and the reconstructed data with 25 points using a Gaussian kernel. Results from various stages of optimization are shown as stems on the *x* axis which are sampled data (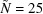) from various stages of optimization. Initially from only integer approximation 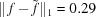 shown in green, after kernel optimization 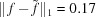 in blue and finally after subdivision optimization 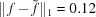 in red.

Let us further illustrate this process on a 1D example. Here the input data consist of 200 points ranging from 0 to 1. The corresponding density function *f* is visualized in Fig. 2 ▸ (plotted in black). The input data are subdivided into 20 equally spaced bins and the number of data points corresponding to each bin is fed to the IA method which reduces the data set to only 25 points. The density function estimated from this downsampled data set is plotted in green in Fig. 2 ▸. At this stage 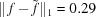 for the 1D example. This 1D example is used in the later sections for the purpose of illustration.

### 2.6. Optimal shape parameter of the kernel function   

**Figure 3:**
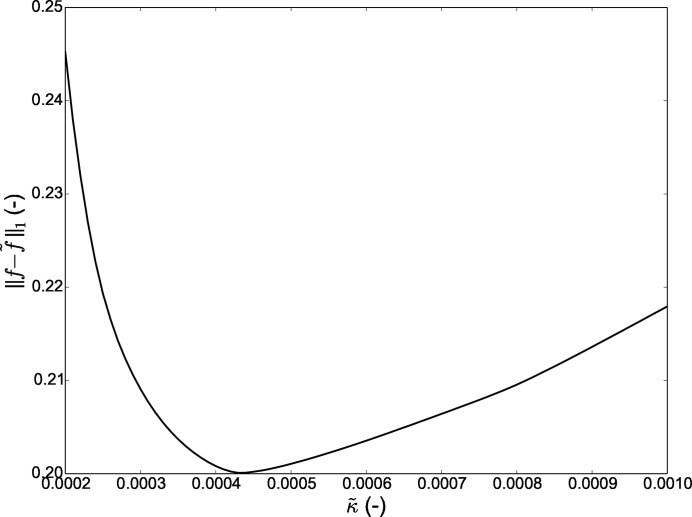
Effect of kernel shape parameter 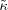 on 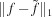 between the input (*f*) and output (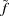) kernel density estimates for the 1D example.

**Figure 4:**
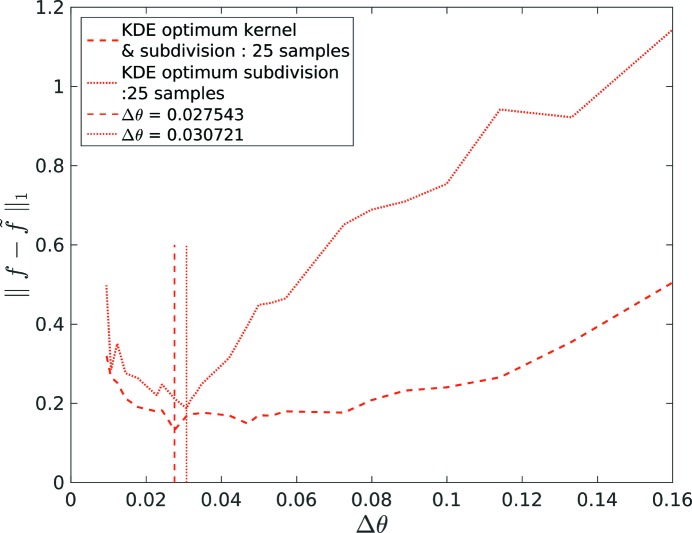
Effect of grid spacing 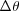 on 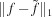 between the input (*f*) and output (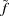) kernel density estimates for the 1D example.

In the above 1D example we observe that the density function recovered from the reduced data set is much too oscillatory. This is because we have chosen the kernel function 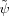 shape parameter 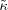 for the reduced data set to be equal to the shape parameter κ of the kernel function ψ that we used for the original data set. However, in general, a smaller sample size requires a broader kernel function. Hence, the objective of this section is to optimize 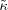 such that the corresponding ODF,
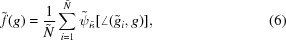
fits best the original ODF,
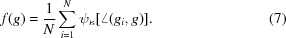



The optimization process of 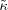 starts with the initial guess equal to κ and progresses in fixed steps selecting the least value of 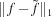. The effect of this optimization for the 1D example is shown in Fig. 3 ▸, in which 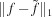 reduces from 0.29 (Fig. 2 ▸) to 0.17 (plotted in blue). The kernel optimization affects the shape of the error function 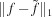 which shifts the minimum value. This results in a different value of optimum 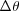, as shown by the curve with a dashed line (with both optimum kernel and optimum subdivision) and the curve with a dotted line (with only optimum subdivision) in Fig. 4 ▸. This comparison justifies the kernel shape optimization in the proposed algorithm.

The results of kernel optimization for various test cases and different 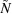 are shown in Table 2 ▸. As explained earlier, these results show that as 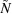 increases the kernel function becomes finer (indicated by smaller values of 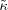).

### 2.7. Optimum grid spacing   

In the previous sections, we have found a subsampling of our input orientations for a given subdivision of the orientation space into cells and an optimal kernel function to recover an ODF 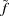. In this section, we aim to optimize the subdivision of the orientation space to obtain an optimal fit between the ODF *f* computed from the input orientations and the ODF 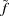 computed from the reduced set of orientations.

Fig. 4 ▸ indicates the dependency of 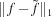 on 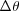 for the 1D problem, and the optimization of 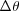 to minimize 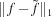 further yields a result of 0.12 shown in Fig. 2 ▸ (plotted as a red line). The optimization procedure is similar to the kernel optimization performed in Section 2.6. The algorithm proceeds in steps of 1 unit of 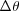 and selects the least value of 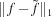 estimated over a range of 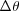.

**Figure 5:**
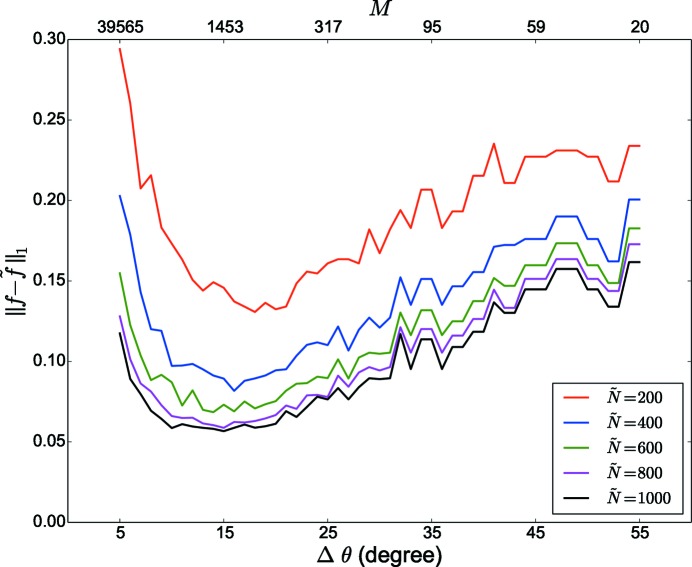
Effect of grid spacing 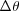 on 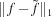 between input (*f*) and output (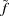) ODFs for the test case of additively manufactured 316L stainless steel produced with a 1000 W laser (316L-1000W), for various 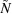 values.

Similarly, the effect of 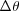 on 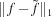 for various 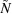 is shown in Fig. 5 ▸ for an actual EBSD data set (test case 316L-1000W).

We observe that the error increases in both directions, *i.e.* if the subdivision becomes too fine or if the subdivision becomes too coarse. On the one hand, if the subdivision becomes too coarse orientations within one cell may have a large misorientation angle and replacing them by their mean value increases the error. On the other hand, if the subdivision is too fine, the integer approximation will result in severe rounding errors.

## 3. Results   

**Figure 6:**
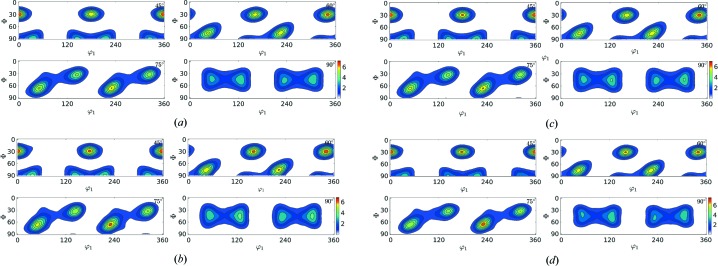
ODF plot comparison of Rolled-Cu from the input orientation set (*N* = 27 000) shown in (*a*) and the reduced orientations 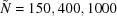 shown in (*b*), (*c*) and (*d*), respectively.

In this section the results of the reconstruction process (Table 3 ▸) are shown in the form of contour plots (Figs. 6 ▸–9 ▸ ▸ ▸) for all of the test cases. The results in Table 3 ▸ show that 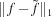 reduces with rising 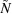 value; however, the corresponding computational time also increases. In these figures, the contour plots of the ODF from input orientations and the reconstructed ODF (for 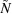 = 150, 400, 1000) are shown. In these contour plots, the peaks and contours in the input ODF are successfully captured by the reconstructed ODF, closely maintaining the intensity of the contours.

**Figure 10:**
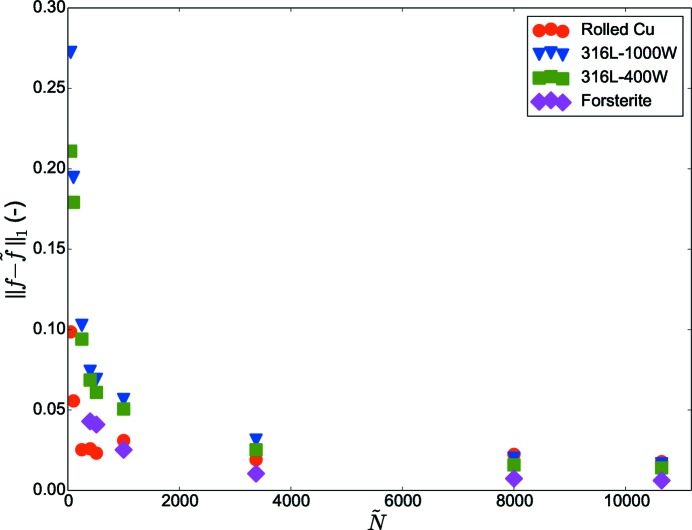
Comparison of 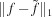 between input (*f*) and output (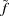) ODFs for the test cases: Rolled-Cu, 316L-1000W, 316L-400W and forsterite calculated for a number of extracted samples.

To further analyze the effect of sample size 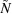 on error 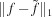, in Fig. 10 ▸ the 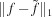 values are compared by varying 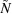 for all test cases. As the value of 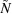 increases, 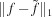 reduces and saturates to an almost constant value. Depending on the input orientations this saturation may be achieved at different values of 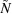.

**Figure 11:**
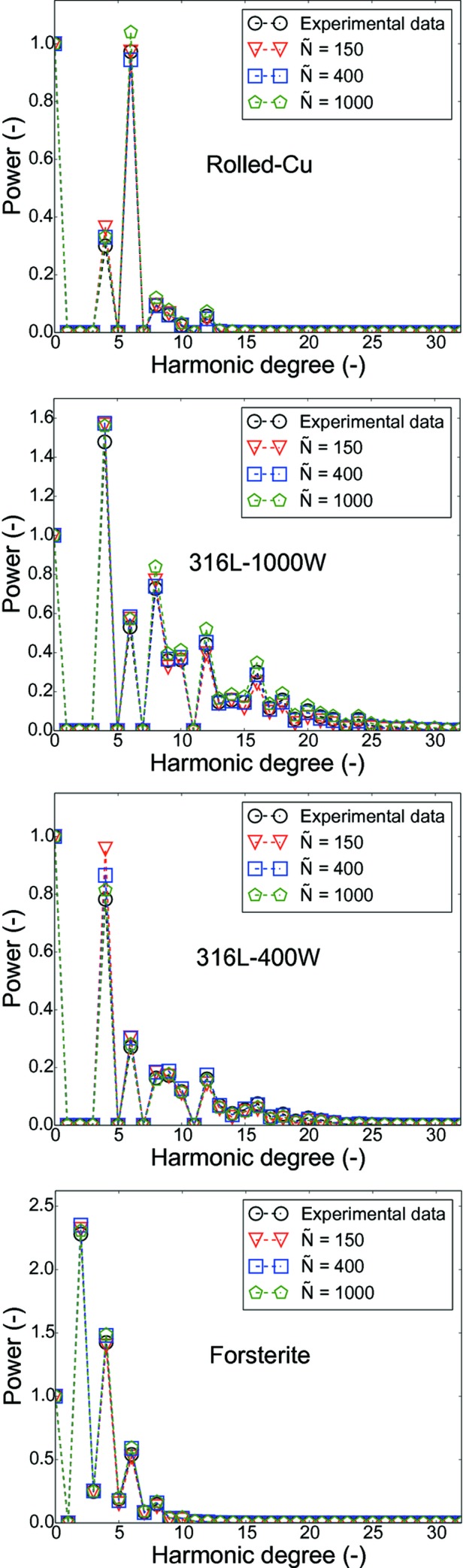
Power plots of ODFs *f* and 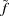 for 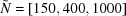 for the test cases: Rolled-Cu, 316L-1000W, 316L-400W and forsterite.

For a more detailed comparison between the ODFs *f* and 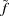 the power plots estimated from the ODFs of the test cases are shown in Fig. 11 ▸. These power plots are estimated by summing the squared Fourier coefficients of a given harmonic order, which shows the contribution of each harmonic order to the texture index; a detailed mathematical description is given in the work of Schaeben *et al.* (2017 ▸). The harmonic contribution from the ODF 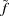 closely matches the value from *f*. This is also observed in Fig. 10 ▸ in which the error 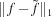 reduces as 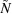 increases.

## 4. Application   

**Figure 12:**
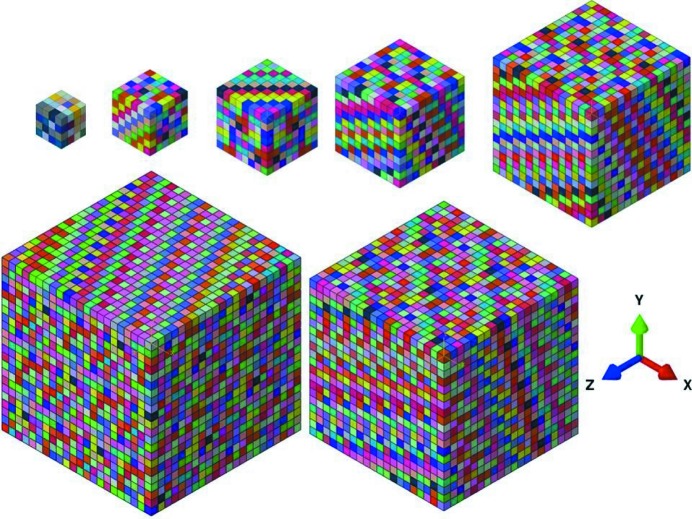
Representative volume elements with 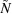 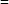 [64, 216, 512, 1000, 3375, 8000] grains. Each grain is represented by one cube corresponding to one finite element.

One of the main applications of this method is numerical modeling like micromechanical modeling. Here, an example of this process is presented for Rolled-Cu. The input is in the form of 27 000 crystallographic orientations. We choose a local crystal plasticity (CP) model without the effect of the strain gradient as described by Ma & Hartmaier (2014 ▸) for numerical modeling of material behavior. The material is assumed to be constructed of periodically repeating volume elements known as the representative volume element (RVE) (refer to Fig. 12 ▸). For details of the applied periodic boundary conditions and homogenization scheme, please refer to Vajragupta *et al.* (2017 ▸).

A virtual uniaxial test is performed for all the RVEs by applying displacement along the *z* or 33 direction. The CP parameters are fitted by comparing the homogenized virtual uniaxial tensile test from the RVE consisting of 27 000 grains with experimental data; therefore the entire input orientation set was used for this test, and no reconstruction process is used for this RVE. Keeping the CP parameters the same, smaller RVEs comprising 64, 216, 512, 1000, 3375, 4913, 8000 and 10 648 grains are generated and corresponding grain crystallographic orientations are generated with the reconstruction algorithm (Table 4 ▸) and hybrid IA method. The results from the smaller RVEs created using both the methods are compared with the reference RVE, *i.e.* the RVE with 27 000 grains.

To exclude the influence of the grain boundary misorientation on the mechanical response of the material, the method for fitting the grain boundary misorientation angle distribution introduced in the work of Biswas *et al.* (2019 ▸) was implemented by using the extracted samples and the RVE geometry; the target distribution followed can be found in the work of Mackenzie (1964 ▸).

Since these orientation sets reconstruct the same input ODF, the output from the virtual tensile test data should be comparable to the results obtained with the model consisting of 27 000 orientations. The finite element method (FEM) simulations are performed with *ABAQUS* (Simulia, 2012 ▸). Each grain in the RVE is discretized using a single C3D8 hexahedral element (Fig. 12 ▸).

**Figure 13:**
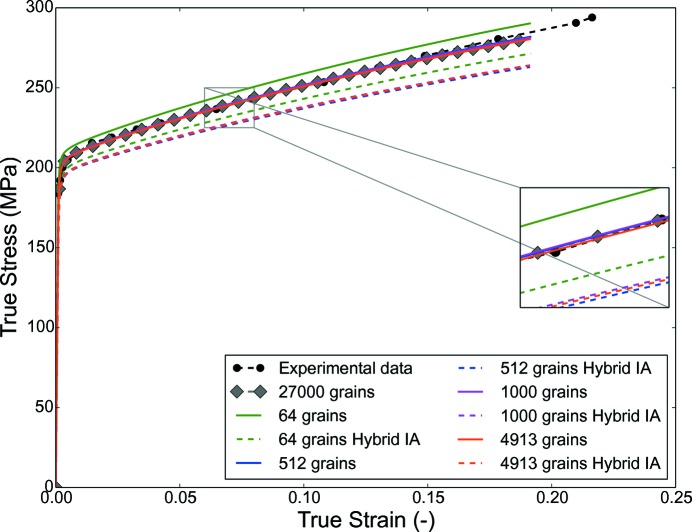
Homogenized CPFEM result comparison for the case of Rolled-Cu for a few selected RVEs constructed using orientations from the reconstruction algorithm and the hybrid IA method; experimental data are obtained by digitizing Fig. 13(*a*) of Knezevic & Landry (2015 ▸).

The homogenized true stress–strain plot shown in Fig. 13 ▸ indicates the difference between the results of the RVEs consisting of discrete orientations generated by the reconstruction algorithm and the hybrid IA method. This mismatch can be attributed to the different input data set. In the case of the hybrid IA, the input is the discrete ODF calculated on predefined SO(3) grid spacing, which may not be optimum for the deterministic IA, whereas the proposed reconstruction algorithm starts with the experimental data set.

Referring to the generated RVEs, the results indicate that the RVE with 216 grains is sufficient to predict the homogenized mechanical properties; a detailed examination of the local stress values (calculated at integration points) gives a different outlook in comparison with the homogenized mechanical properties. The local stress values can be important for modeling phenomena like fracture, damage *etc*.

**Figure 14:**
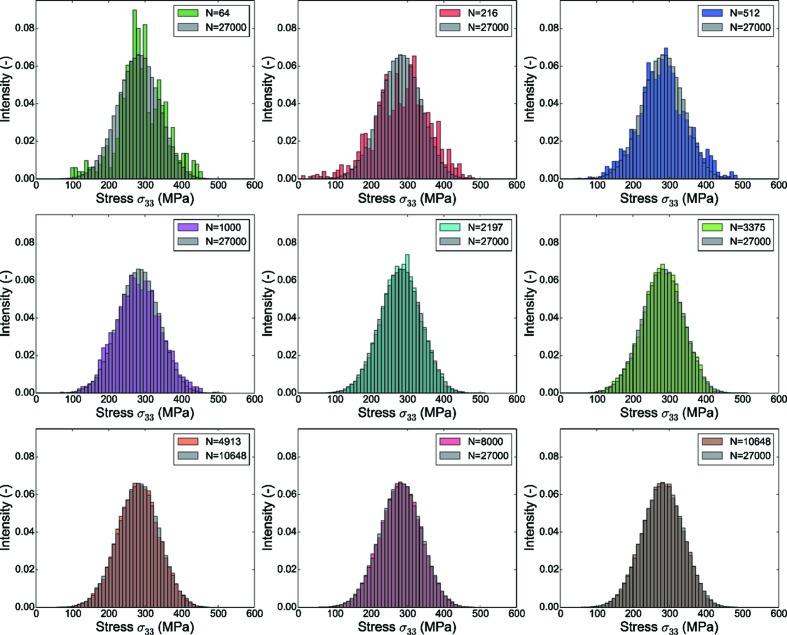
Comparison of stress components along the tensile load direction (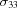) (distribution in the form of a histogram) for RVEs with 64, 216, 512, 1000, 3375, 4913, 8000 and 10 648 grains with the reference RVE (27 000 grains). The bin width of the histogram is 10 MPa.

**Figure 15:**
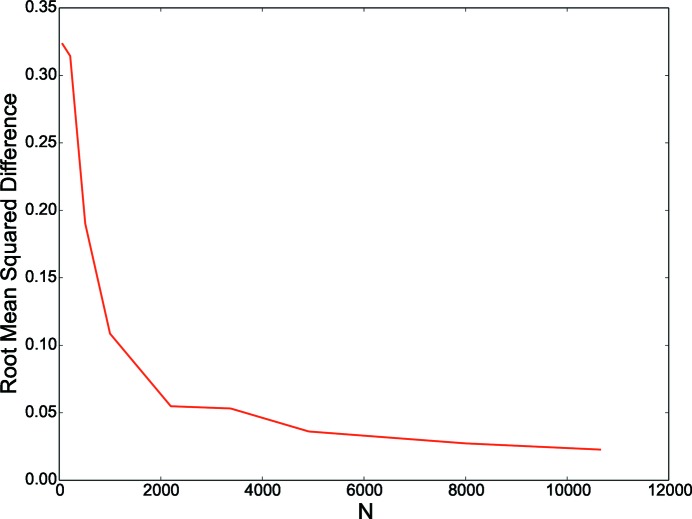
Root-mean-square difference between the stress distribution from the reference RVE (27 000 grains) and smaller RVEs consisting of 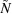 = [64, 216, 512, 1000, 2197, 3375, 4913, 8000, 10 648].

The distribution of the local stress values in smaller RVEs is shown in Fig. 14 ▸ and compared with the local stress distribution in the reference RVE, and the difference is illustrated in Fig. 15 ▸ as the root-mean-square difference. Evidently, as the number of grains in the RVE increases, the difference between the stress distribution from the smaller RVE and the reference RVE also decreases. These results indicate that the value 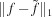 only specifies a minimum requirement, and the strategy for numerical modeling should be decided by the objective of modeling. If the objective in this test case (Rolled-Cu) is to model damage, perhaps an RVE with a minimum of 1000 grains should be considered.

Another important aspect of textured materials is the anisotropy in material properties. This study is performed for a hypothetical test case of copper with fiber texture in which the 110 fiber is parallel to the *Y* direction of the RVE. The CPFEM parameters are the same as those in the work of Anand (2004 ▸) and the RVEs with *N* = [216, 512, 1000, 10 648] are similar to those shown in Fig. 12 ▸. The boundary conditions are similar to the previous test case of Rolled-Cu. However, in this test case, the uniaxial tensile test is performed in all three sample directions for all the RVEs to study the anisotropy in homogenized stress–strain behavior. The input orientation consists of 500 000 orientations generated analytically using *MTEX* (Bachmann *et al.*, 2010 ▸); the results of the reconstruction process are shown in Table 5 ▸.

**Figure 16:**
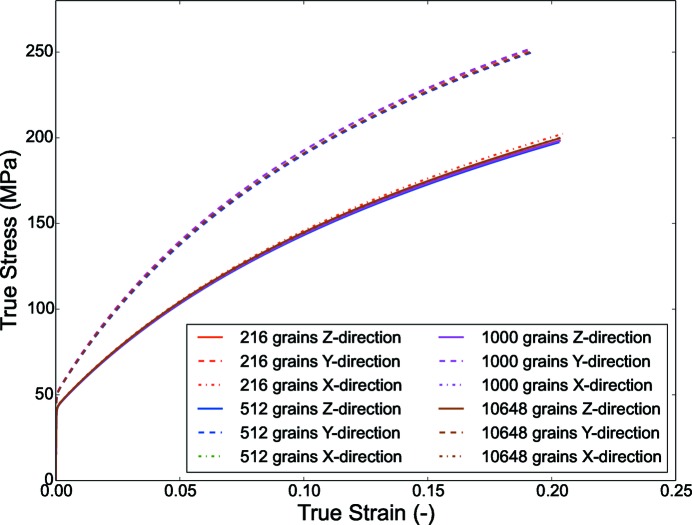
Homogenized CPFEM result comparison for the case of copper with fiber texture.

Fig. 16 ▸ shows the comparison between the stress–strain plot of the RVEs. Since the 110 fiber is aligned along the *Y* direction the stress values are much higher than in the other two directions. All the RVEs show a similar stress–strain behavior. This indicates that the extracted samples can successfully reconstruct the input ODF and are also able to capture the mechanical behavior.

## 5. Conclusion   

In this work, an algorithm for the reconstruction of the ODF from an EBSD experiment by a set of equally weighted orientations has been proposed. It is based on the deterministic integer approximation method introduced by Leffers & Jensen (1986 ▸), but the previously reported problem of overweighting is tackled by optimizing the SO(3) grid and kernel function used for the reconstruction. The quality of the reconstruction is judged not only by the *L* ^1^ norm of the difference between the input and the reconstructed ODF but also by the ODF power plot. The application of this method in the prediction of mechanical behavior using CPFEM provides further insight into the importance of precise representation of the reduced orientation set in CPFEM simulations. This study shows that the *L* ^1^ norm of the difference between the input and the reconstructed ODF estimated from the sample orientations gives only a minimum criterion for the number of samples to be incorporated in the RVE. However, the influence of sample size on the local mechanical output like stress can be observed and should be considered during micromechanical modeling. In addition to this, we also demonstrate the ability of the reduced orientation set to predict anisotropy in yield strength and hardening behavior, through micromechanical simulations of the RVE with fiber texture. This case study shows a good agreement for the prediction of these mechanical behaviors between the various sample sizes ranging from 216 to 10 648 orientations.

## Figures and Tables

**Figure 7:**
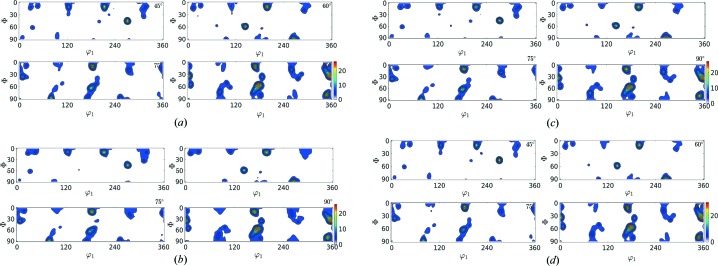
ODF plot comparison of 316L-1000W from the input orientation set (*N* = 118 848) shown in (*a*) and the reduced orientations 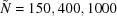 shown in (*b*), (*c*) and (*d*), respectively.

**Figure 8:**
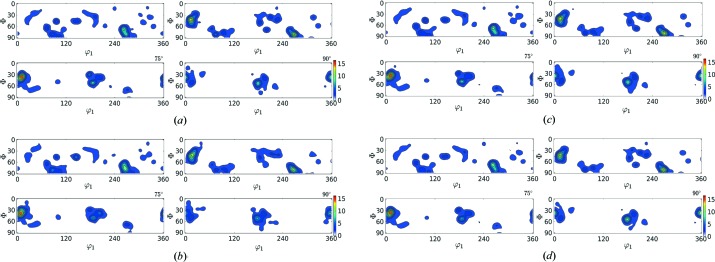
ODF plot comparison of 316L-400W from the input orientation set (*N* = 46 310) shown in (*a*) and the reduced orientations 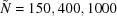 shown in (*b*), (*c*) and (*d*), respectively.

**Figure 9:**
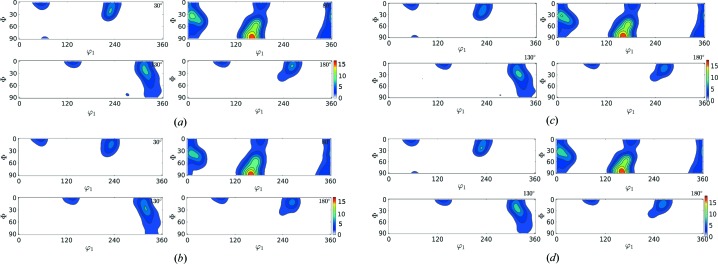
ODF plot comparison of forsterite from the input orientation set (*N* = 152 345) shown in (*a*) and the reduced orientations 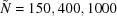 shown in (*b*), (*c*) and (*d*), respectively.

**Table 1:** Inventory of symbols and notations

*N*	Number of input orientations
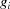 , 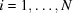	Input orientations
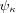	Kernel function used for density estimation from the input orientations
*f*	Orientation density function estimated from the input orientations
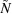	Target number of orientations of the reduced set
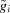 , 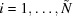	Reduced set of orientations
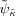	Kernel function used for density estimation from the reduced set of orientations
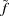	Orientation density function estimated from the reduced set of orientations
*M*	Number of cells of the subdivision of the orientation space
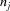 , 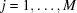	Number of input orientations that fall into each of the cells
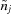 , 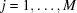	Number of reduced orientations that fall into each of the cells
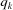 , 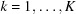	Equally spaced grid of orientations used for estimating the error between two ODFs

**Table 2:** 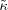
 optimization result for various test cases

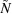	216	400	512	1000	2197	3375	4913	8000	10648
Rolled-Cu (κ = 10°)	11°	11°	10.5°	10°	10°	10°	10°	10°	10°
316L-1000W (κ = 4.7°)	5.7°	5.2°	5.2°	4.7°	4.7°	4.7°	4.7°	4.7°	4.7°
316L-400W (κ = 5.9°)	6.4°	6.4°	6.4°	6.4°	5.9°	5.9°	5.9°	5.9°	5.9°
Forsterite (κ = 11.9°)	13.4°	12.4°	11.9°	11.9°	11.9°	11.9°	11.9°	11.9°	11.9°

**Table 3:** Application of the reconstruction algorithm to various test cases The values shown in the table are 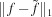 and the values within parentheses show computation time in seconds.

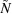	Rolled-Cu	316L-1000W	316L-400W	Forsterite
400	0.0221 (176)	0.0715 (82)	0.0645 (119)	0.0442 (80)
512	0.0183 (189)	0.0618 (107)	0.0557 (153)	0.0383 (120)
1000	0.0217 (221)	0.0545 (173)	0.0466 (201)	0.0206 (140)
3375	0.0117 (309)	0.0267 (182)	0.0236 (275)	0.0071 (154)
8000	0.0074 (504)	0.0201 (248)	0.0135 (541)	0.0046 (262)
10648	0.0082 (699)	0.0169 (324)	0.0120 (669)	0.0038 (319)

**Table 4:** ODF reconstruction data for Rolled-Cu

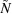	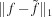
64	0.07760
216	0.03458
512	0.02317
1000	0.03100
2197	0.02387
3375	0.01911
4913	0.01522
8000	0.02242
10648	0.01788

**Table 5:** ODF reconstruction data for fiber texture (110 fiber parallel to the *Y* direction)

Orientation samples	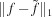
216	0.01498
512	0.01131
1000	0.00605
10648	0.00019
